# Semiconducting Polymer Nanoporous Thin Films as a Tool to Regulate Intracellular ROS Balance in Endothelial Cells

**DOI:** 10.1021/acsami.3c06633

**Published:** 2023-07-19

**Authors:** Miryam Criado-Gonzalez, Luca Bondi, Camilla Marzuoli, Edgar Gutierrez-Fernandez, Gabriele Tullii, Carlotta Ronchi, Elena Gabirondo, Haritz Sardon, Stefania Rapino, Marco Malferrari, Tobias Cramer, Maria Rosa Antognazza, David Mecerreyes

**Affiliations:** † POLYMAT, University of the Basque Country UPV/EHU, Paseo Manuel de Lardizabal 3, 20018 Donostia-San Sebastián, Spain; ‡ Department of Physics and Astronomy, University of Bologna, Viale Carlo Berti Pichat 6/2, 40127 Bologna, Italy; § Center for Nano Science and Technology@PoliMi, Istituto Italiano di Tecnologia, Via Raffaele Rubattino 81, 20134 Milano, Italy; ∥ Dipartimento di Fisica, Politecnico di Milano, Piazza Leonardo da Vinci 32, 20133 Milano, Italy; ⊥ XMaS/BM28-ESRF, 71 Avenue Des Martyrs, F-38043 Grenoble Cedex, France; # Department of Physics, University of Warwick, Gibbet Hill Road, Coventry CV4 7AL, U.K.; ¶ Department of Chemistry “Giacomo Ciamician”, University of Bologna, 40126 Bologna, Italy; ∇ Ikerbasque, Basque Foundation for Science, 48013 Bilbao, Spain

**Keywords:** poly(3-hexylthiophene), porous films, biophotonics, reactive oxygen species (ROS), cell optical modulation

## Abstract

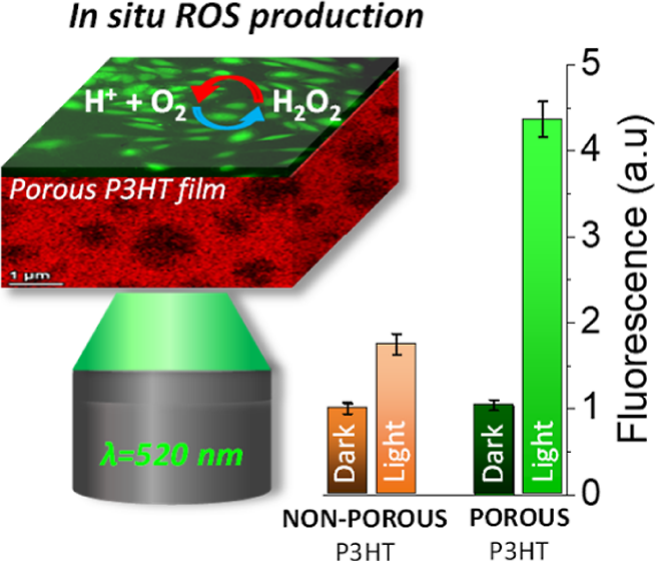

The design of soft and nanometer-scale photoelectrodes able to stimulate and promote the intracellular concentration of reactive oxygen species (ROS) is searched for redox medicine applications. In this work, we show semiconducting polymer porous thin films with an enhanced photoelectrochemical generation of ROS in human umbilical vein endothelial cells (HUVECs). To achieve that aim, we synthesized graft copolymers, made of poly(3-hexylthiophene) (P3HT) and degradable poly(lactic acid) (PLA) segments, P3HT-*g*-PLA. In a second step, the hydrolysis of sacrificial PLA leads to nanometer-scale porous P3HT thin films. The pore sizes in the nm regime (220–1200 nm) were controlled by the copolymer composition and the structural arrangement of the copolymers during the film formation, as determined by atomic force microscopy (AFM) and transmission electron microscopy (TEM). The porous P3HT thin films showed enhanced photofaradaic behavior, generating a higher concentration of ROS in comparison to non-porous P3HT films, as determined by scanning electrochemical microscopy (SECM) measurements. The exogenous ROS production was able to modulate the intracellular ROS concentration in HUVECs at non-toxic levels, thus affecting the physiological functions of cells. Results presented in this work provide an important step forward in the development of new tools for precise, on-demand, and non-invasive modulation of intracellular ROS species and may be potentially extended to many other physiological or pathological cell models.

## 1 Introduction

In the last few decades, reactive oxygen species (ROS) have emerged as a highly powerful cell signaling agent, and many recent studies have elucidated the key role of oxidants (*i.e.*, superoxide anions, hydroxyl radicals, and the more stable hydrogen peroxide, among others) not only in disease but also in physiology.^1^ ROS effect can vary from detrimental, with harmful effects on cell viability, determining cell apoptosis, and serious tissue damage, to highly beneficial in most biological processes, including cell differentiation, proliferation, and migration, up to specific functionality.^2,3^ Redox biology is indeed ubiquitous in virtually all cell types and biological systems, covering a broad range of medical issues such as inflammation, cardiovascular diseases, cancer, muscle biology, renal function, airway diseases, and neuroscience.^4^ Therefore, the concept of “redox medicine” has gained increasing attention as a promising and powerful approach to finely regulate both eustress and distress conditions, *i.e.*, to target both physiological and pathophysiological conditions.^4,5^ Not surprisingly, the watershed between the two has been first identified in intracellular ROS concentration.^6,7^ Although redox materials are used for the prevention and treatment of various diseases in the preclinical stage, translating therapeutic redox materials to the clinic is still evolving.^8,9^

ROS can be produced endogenously, from an incomplete reduction of oxygen and the enzyme nicotinamide adenine dinucleotide phosphate oxidase in the plasma membrane, or through exogenous stimuli like UV light, ionizing radiation, or xenobiotics.^10^ While endogenous biochemical approaches are effective to target cellular redox signaling processes, they are irreversible and present limited spatiotemporal resolution.^11−13^ These drawbacks can be overcome by using physical stimuli, *i.e.*, light, heat, and electricity.^14,15^ The use of light radiation, in particular, is expected to provide lower invasiveness, relying on wireless stimulation, reversibility, and higher spatial selectivity than electrical, thermal, and pharmaceutical methods.^16−18^ However, its application is still limited by its low efficacy and somewhat erratic results obtained in different cell and tissue models.^19,20^ Most importantly, the use of bare photoexcitation does not allow for the necessary fine-tuning of ROS concentration, being limited to fixed power density ranges identified as safe regimes for photostimulation.^21^

In this scenario, the development of biocompatible and photoactive materials capable of finely and reliably tuning intracellular ROS concentrations is therefore of high interest. Among other possibilities, organic semiconductors represent a promising strategy due to their excellent optical and electronic properties, highly versatile fabrication technologies, biocompatibility, and compliance with *in vitro* and *in vivo* operations. Semiconducting polymers, which have been widely used for decades in optoelectronics, *i.e.*, organic photovoltaics,^22^ organic light-emitting diodes,^23^ or organic field-effect transistors,^24^ have found increasing interest in photo-actuated biophotonics.^25,26^ Apart from their intrinsic conductivity and optical properties, the chemical versatility, biocompatibility, and flexibility of conjugated polymers make them ideal candidates to accomplish the multifunctional properties requested for cell–material interfaces and, in particular, for the control of localized photoelectrochemical reactions.^27,28^ Poly(3-hexylthiophene) (P3HT) is a p-type polymer with interesting optoelectronic properties that acts as an efficient and highly biocompatible phototransducer, able to trigger biological pathways relevant to cardiac repair with a minimally invasive and gene-less approach.^29,30^ When used as a photocathode in an aqueous environment, oxygen is the main acceptor,^31^ and it generates H_2_O_2_ and other intermediate ROS species on the semiconductor surface, which can act as chemical signals for the cellular environment.^1,32^ Semiconducting polymer nanomaterials based on P3HT have been shown to interact functionally with living cells and generate ROS upon visible light irradiation to trigger intracellular calcium ion flux or induce redox signaling processes.^33−38^ In particular, P3HT-based thin films exhibit a stable and efficient photocatalytic activity in aqueous environments,^39^ making them suitable as extracellular bio-photoelectrodes to induce redox signaling processes.^40−42^ Even more importantly, the material does not show relevant, irreversible photodegradation effects over long-term *in vitro* and *in vivo* functionality and allows for precise, on-demand targeting of subcellular organelles.^43,44^

From the abovementioned study cases, it clearly emerges how the structural engineering of the semiconducting polymer material, influenced by both chemical and physical parameters, plays a key role in the modulation of photophysical processes and intracellular ROS concentration, and it will ultimately determine their most successful applications in the redox medicine field.^45,46^ However, *in vivo* photostimulation as a clinical treatment approach suffers from low photoelectrochemical yield due to the significant absorption of optical excitation by skin layers and the thermalization of the absorbed energy.^47^ To address this limitation, the development of materials with a smaller bandgap or nanomaterials with better photon-to-ROS conversion yield is necessary and actively searched. Diverse approaches have already been explored to achieve a higher interfacial area, such as structuring the surface of P3HT films with micropillars that also provided a mechanically compliant environment and close contact with neuronal cells,^48^ or by using polystyrene (PS) spheres as removable molds.^49^

In this work, we present nanoporous P3HT thin films with a superior ability for generating ROS through photostimulation, as they enhanced the photoreducing capabilities of the biophotonic device and increased light absorption through scattering due to the surface area increase conferred by the tunable porosity. The nanoporous P3HT thin films were prepared by selective hydrolysis of the sacrificial poly(lactic acid) (PLA) segment of a new family of P3HT-*g*-PLA graft copolymers. The relationship between the pore size, morphology, and copolymer molecular composition was investigated, and the influence of the porosity on the photocurrent properties was explored. Finally, the effective employment of these porous thin films as biophotonic devices to optically wireless modulate intracellular ROS production was demonstrated. To this goal, we selected human umbilical vein endothelial cells (HUVECs) as a biologically relevant model for the endothelium function. The regulation of vascular processes, in particular angiogenesis, is fundamental for the treatment of many diseases, such as cardiovascular pathologies and cancer, and it is strictly connected to the modulation of intracellular ROS concentration.^1,50,51^

## 2 Results and Discussion

### 2.1 Synthesis and Characterization of P3HT Porous Films

**Figure 1:**
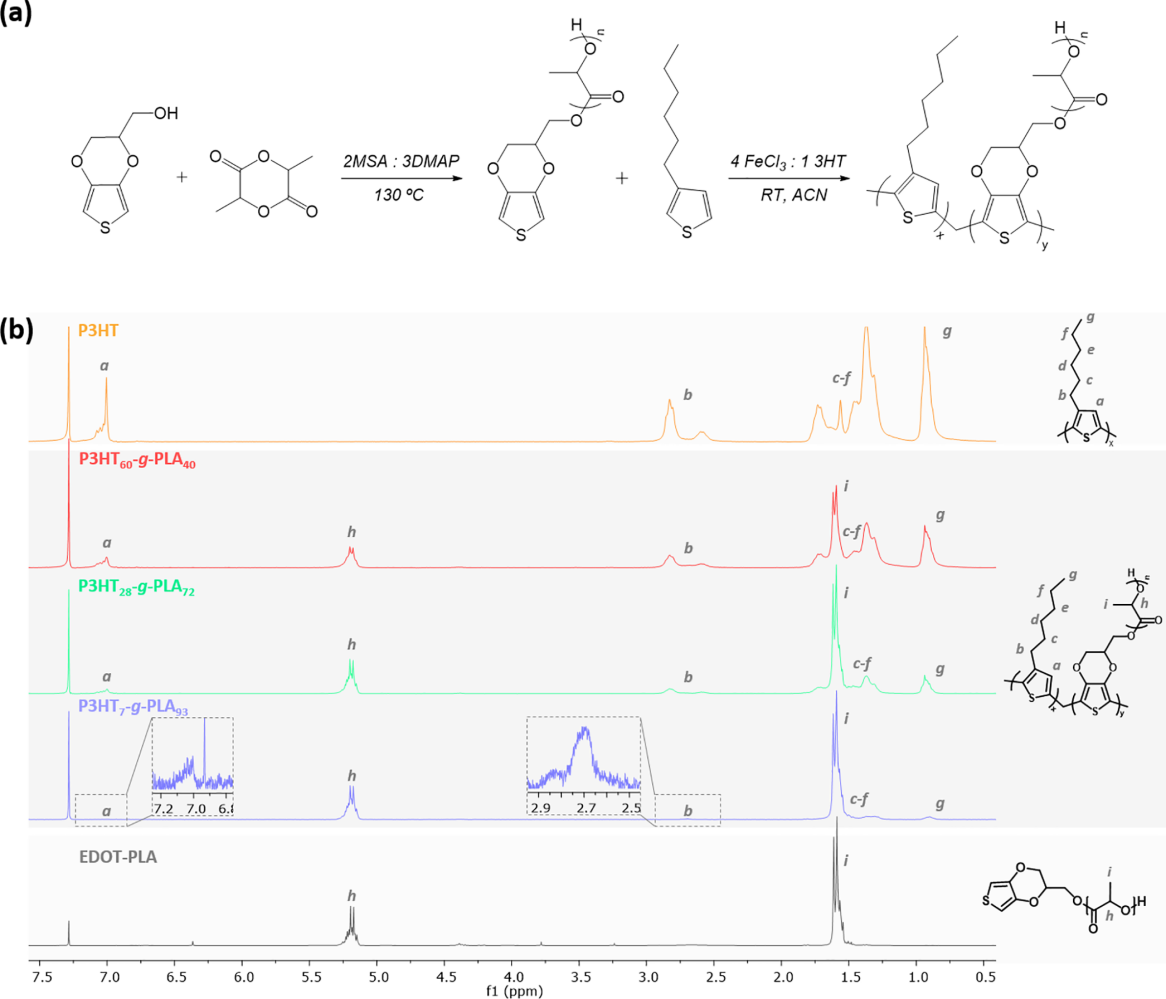
(a) Chemical routes employed to synthesize the α-EDOT-PLA macromonomer by ROP, followed by the synthesis of P3HT-*g*-PLA copolymers by chemical oxidative polymerization. (b) ^1^H NMR spectra of the synthesized macromonomer α-EDOT-PLA, homopolymer P3HT, and graft copolymers P3HT-*g*-PLA.

In order to prepare semiconducting polymer nanoporous thin films, we investigated the well-known method of selective hydrolysis or etching of one of the segments of a block or graft copolymer.^52^ This methodology is based on the phase separation of the block copolymers in the nanometer regime and the easy hydrolysis of the polyester segments, such as polylactide or poly(ε-caprolactone), well studied by Hillmyer and Grande *et al.*(53−55) This method, widely applied to obtain nanoporous polymers of PS or poly(acrylates), was also applied to the development of ordered nanoscale morphologies consisting of self-assembled P3HT donor domains of molecular dimension, each of them separated by fullerene C_60_ hydroxide acceptor domains, to be used in the fabrication of idealized bulk heterojunctions for organic/hybrid solar energy devices.^56,57^ However, to the best of our knowledge, nanoporous semiconducting polymers have not been previously employed for biophotonics devices. For this purpose, we first designed a synthetic strategy for a P3HT graft copolymer. Thus, P3HT-*g*-PLA copolymers were synthesized by graft copolymerization of 3-hexylthiophene (3HT) and a thiophene α-EDOT-PLA macromonomer. In more detail, the α-EDOT-PLA macromonomer was first synthesized by organocatalyzed ring-opening polymerization (ROP) using EDOT-methanol as the chain initiator of the lactide polymerization (Figure 1a). The ROP was carried out in bulk using an organocatalyst formed by a mixture of methanesulfonic acid (MSA) and 4-dimethylaminopyridine (DMAP) in a ratio of 2:3, which was previously reported for the synthesis of poly(l-lactide) (PLA) using benzyl alcohol as initiator.^58^ After a 75 min reaction at 130 °C, a 90% conversion was reached as calculated by ^1^H NMR (Figure 1b). The molecular weight of the α-EDOT-PLA macromonomer was *M*_n_ = 9400 g mol^–1^ as measured by gel permeation chromatography (GPC). Then, P3HT-*g*-PLA copolymers were successfully synthesized by chemical oxidative polymerization of 3HT and the previously synthesized α-EDOT-PLA macromonomer in the presence of an excess of FeCl_3_, leading to dark brown dispersions. P3HT-*g*-PLA copolymers with different PLA compositions were synthesized by varying the initial 3HT/PLA ratio in the reaction. Yields higher than 40% were obtained, and the proportion of both components in the graft copolymers was determined by ^1^H NMR (Figure 1b). Results showed a peak at 0.9 ppm, attributed to −CH_3_ of P3HT,^59^ and another peak at 5.2 ppm due to the protons present on the backbone of the repeating units of PLA.^58^ The ratio between the integrated signals of P3HT and PLA allowed us to determine the final molar composition of each component in the graft copolymers (Table 1). It should be noted that the high molecular weights of the P3HT-*g*-PLA copolymers and the monomodal size exclusion chromatography (SEC) distributions allow us to state that no residual macromonomer is present (Figure S1).

**Table 1:** Chemical Oxidative Polymerization of 3HT Itself and with the α-EDOT-PLA Macromonomer in Acetonitrile at Room Temperature Overnight

code	P3HT (% mol)^a^	yield (% wt)	P3HT (% mol)^b^	PLA (% mol)^b^	*M*_n_ (g mol^–1^)^c^	*D*^d^	synthesized polymer
1	100	43	100		9,300	4.79	P3HT
2	46	54	60	40	16,300	2.69	P3HT_60_-*g*-PLA_40_
3	31	40	28	72	24,000	1.80	P3HT_28_-*g*-PLA_72_
4	21	45	7	93	29,500	1.98	P3HT_7_-*g*-PLA_93_

a Mol fraction of 3HT in the reaction feed.

b Mol fraction of P3HT and PLA in the copolymers calculated by ^1^H NMR.

c *M*_n_ of P3HT-*g*-PLA copolymers determined by using PS standards.

d Dispersity (*D*) = *M*_w_/*M*_n_ calculated by SEC.

**Figure 2:**
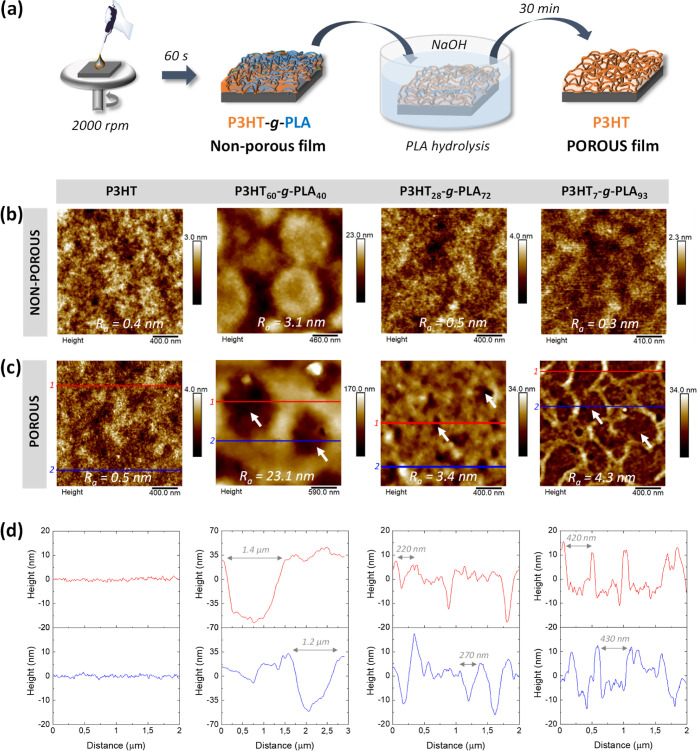
(a) Schematic representation of the fabrication of non-porous thin films by spin coating, followed by a hydrolysis step of the PLA grafts to obtain porous thin films. Topographical AFM images of the (b) non-porous films and (c) porous films after PLA hydrolysis. The white arrows indicate the places where some holes are located. Red (1) and blue (2) lines show the cut-positions to determine the pore dimensions in (d).

Synthesized polymers 1–4, as listed in Table 1, were solved in chlorobenzene and spin-coated on top of indium–tin-oxide (ITO)-glass substrates in order to obtain compact thin films. In the next step, porous thin films were obtained by PLA hydrolysis in the presence of NaOH 0.5 M (Figure 2a). The efficacy and reliability of PLA hydrolysis in inducing porosity were corroborated by ^1^H NMR analysis (Figure S2). Before PLA hydrolysis, non-porous films show the characteristic peaks of P3HT and PLA; after NaOH treatment, the PLA component is totally hydrolyzed, and the remaining spectrum shows the characteristic features of the P3HT component only. The molecular weight after PLA hydrolysis was also determined by GPC (Figure S1 and Table S1). The results showed a displacement to lower molecular weights after the PLA hydrolysis, as pointed out by the lower hydrodynamic volume, being this displacement stepper as the percentage of PLA in the graft copolymer increased with a decrease of the polymerization degree of the thiophene unit.

All films show nanometer-scale thicknesses (200–250 nm), which decrease after PLA hydrolysis, being this reduction more pronounced at higher PLA concentrations in the graft copolymer (Figure S3). The morphology of the films was then characterized by atomic force microscopy (AFM) (Figure 2b). Non-porous (*i.e.*, prior PLA hydrolysis) films prepared with the copolymers P3HT_7_-*g*-PLA_93_ and P3HT_28_-*g*-PLA_72_ display a surface topography similar to those prepared with the homopolymer P3HT, used as a reference control, and are characterized by a surface roughness *R*_a_ ≤ 0.5 nm. Films corresponding to the graft copolymer P3HT_60_-*g*-PLA_40_ exhibit a totally different surface topography, with round domains surrounded by a continuous phase and a higher surface roughness (*R*_a_ = 3.1 nm). It is known that micro-phase separation in copolymers is influenced by different aspects like composition, copolymer architecture, or crystallinity. This is even more evident in the case of graft copolymers, where one of the polymer blocks is composed of a conducting or semiconducting polymer. In particular, we have a semicrystalline graft copolymer where we observe the crystallinity of both P3HT and PLA, as proven by the grazing-incidence wide-angle X-ray scattering (GIWAXS) analysis (Figure S4a), which will complicate the system and could be a reason for the domain size dependence on the composition. This is in agreement with previous works, in which graft copolymers made of the conducting polymer poly(3,4-ethylenedioxythiophene) (PEDOT) and poly(ethylene glycol methyl ether methacrylate) (POEGMA), PEDOT-*g*-POEGMA, exhibited a non-sequencial phase separation with the different copolymer compositions, where a different morphology was observed between PEDOT-*g*-POEGMA with mass ratios of 20:80 and 50:50, whereas non-significant differences were observed between mass ratios of 50:50 and 80:20.^60^ Similar findings were also observed in the case of P3HT and phenyl-C_61_-butyric acid methyl ester.^61^ After PLA hydrolysis, P3HT films show, as expected, the same morphology and surface roughness (Figure 2c, left panel). Conversely, the films prepared from the graft copolymers P3HT-*g*-PLA display substantial changes, with the appearance of pores, evidenced as dark areas in the AFM micrographs (Figure 2c). Besides, the surface roughness considerably increases, reaching values up to *R*_a_ = 23.1 nm as well as the surface area (*S*_a_) (Figure S5). The dimensions of the pores were determined by cutting edges in two different representative areas of the micrographs (Figure 2d). In P3HT_7_-*g*-PLA_93_, the main PLA component of the copolymer is hydrolyzed, thus creating the intermediate large pores (∼420 nm diameter and ∼20 nm height) and the smallest increase of the surface area (104%). Upon decreasing the PLA percentage, P3HT_28_-*g*-PLA_72_ films exhibit smaller pores with ∼220 nm diameter and ∼15 nm height, providing the largest increase of the surface area (168%). Surprisingly, P3HT_60_-*g*-PLA_40_ films show the largest pores (∼1.2 μm diameter and ∼100 nm height), possibly originated by the different surface morphology and co-existence of both graft copolymer components during the thin film preparation, with a 112% increase of *S*_a_. As a control, thin films were prepared with the homopolymer PLA; as expected, they were completely hydrolyzed after NaOH treatment (Figure S6).

**Figure 3:**
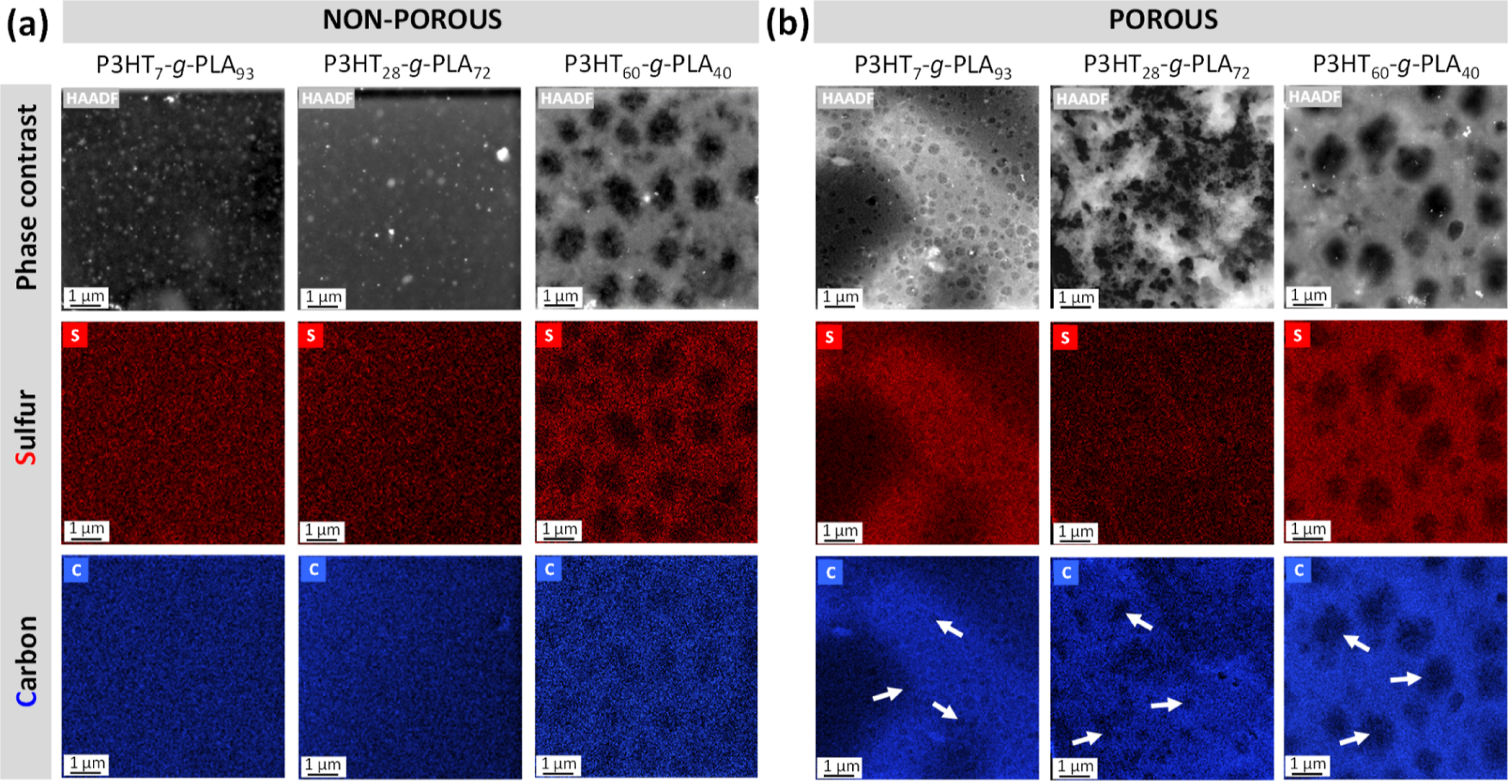
TEM images, including the EDX analysis showing sulfur atoms in red and carbon atoms in blue, of the (a) non-porous films, and (b) porous films after PLA hydrolysis. The white arrows indicate the formation of pores.

To delve into the distribution of both components, P3HT and PLA, in the thin films, transmission electron microscopy (TEM) with energy dispersive X-ray analysis (EDX) was performed (Figure 3). Sulfur atoms present in the P3HT are shown in red color, whereas carbon atoms present in both P3HT and PLA are shown in blue. In the case of non-porous thin films (Figure 3a), those prepared with graft copolymers with higher PLA proportions, *i.e.*, P3HT_7_-*g*-PLA_93_ and P3HT_28_-*g*-PLA_72_, show a homogeneous distribution of sulfur and carbon atoms throughout the whole surface area. Nevertheless, thin films prepared with the copolymer P3HT_60_-*g*-PLA_40_ with a higher P3HT proportion exhibit phase-separated domains between both components, with PLA domains of ∼1 μm diameter surrounded by contiguously conductive P3HT. After PLA hydrolysis, the red color of sulfur atoms present in the P3HT remains stable, keeping the same distribution and appearance in the thin films, whereas the blue color intensity of carbon atoms in the PLA domains disappears, as highlighted by the white arrows (Figure 3b), in agreement with the formation of the pores with ∼1 μm diameter as evidenced by AFM (Figure 2c,d).

It is well known that hydrophilicity is a key factor in material interactions with aqueous electrolytes and cells.^62,63^ Thus, the water contact angle (WCA) of the P3HT films was measured (Figure S7). Non-porous films formed with P3HT and the graft copolymers P3HT-*g*-PLA are hydrophobic with WCA values of ∼100°, whereas PLA films are less hydrophobic with WCA of ∼80°. The WCA of P3HT films used as control did not change after PLA treatment. A similar finding is observed in the case of the copolymer with the highest P3HT percentage, P3HT_60_-*g*-PLA_40_. These results are in agreement with reported WCA values for P3HT^64^ and PLA^65^ films. However, WCA decreases up to ∼30° for porous films prepared with lower P3HT percentages, P3HT_7_-*g*-PLA_93_ and P3HT_28_-*g*-PLA_72_, proving that the porosity and the NaOH treatment confer hydrophilic properties to the films due to maximized contact with aqueous electrolytes such as phosphate buffer saline (PBS) and cell cultures.

### 2.2 Optical and Photoelectrochemical Properties of Non-Porous and Porous Thin Films

**Figure 4:**
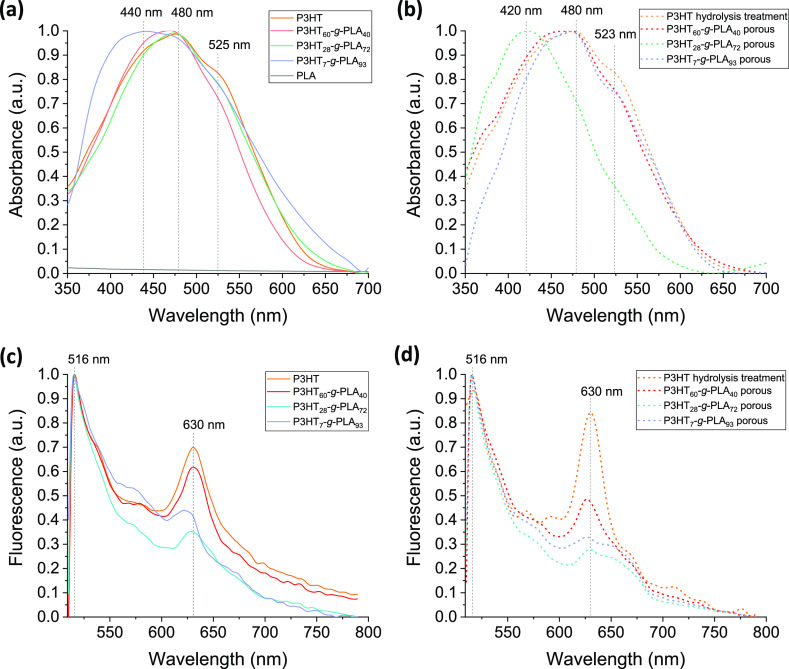
Normalized optical absorption spectra of (a) non-porous (solid curves) and (b) porous (dashed curves) thin films. Normalized fluorescence spectra (λ_exc_ = 480 nm) of (c) non-porous (solid curves) and (d) porous (dashed curves) thin films.

The optical absorption properties of the films were determined by UV–vis spectrophotometry (Figure 4a,b). The UV–vis spectrum of P3HT shows an absorption peak at 480 nm due to the π–π* electronic transition of P3HT chains in a flexible random-coil conformation,^66^ and a peak at 525 nm attributed to increased planarity of P3HT backbones.^66^ In the case of the graft copolymers, there is a blue shift and broadening of the maximum absorption peak, up to 440 nm for P3HT_7_-*g*-PLA_93_, as the percentage of PLA in the graft copolymers increases, which is coherent with a reduced average conjugation length of the P3HT segments. As the PLA content increases, the reduced P3HT segments cannot crystallize as efficient as the homopolymer.^67^ This is corroborated by GIWAXS measurements (Figure S4a). The pattern from the bare P3HT shows characteristic reflections associated to this polymer and its semicrystalline nature. The presence of EDOT-PLA segments in the segments reduces the conjugation length of P3HT segments and, therefore, their crystallinity degree, as confirmed by the disappearance of P3HT reflections in the copolymer GIWAXS patterns. The NaOH treatment used to induce the PLA hydrolysis does not sizably impact the optical spectra (Figure 4b, dashed orange curve) and the crystalline structure (Figure S4b) of P3HT homopolymer films. However, porosity induces important changes in the copolymers’ absorbance, depending in turn on the composition. In more detail, the UV–vis absorption spectra of porous thin films with larger pore sizes, P3HT_60_-*g*-PLA_40_ (∼1.2 μm diameter and ∼100 nm height) and P3HT_7_-*g*-PLA_93_ (∼420 nm diameter and ∼20 nm height) (Figure 2c,d), exhibit a maximum of absorbance at 480 nm, while P3HT_28_-*g*-PLA_72_ porous thin films with the smallest pore sizes, ∼220 nm diameter and ∼15 nm height (Figure 2c,d), show a maximum of absorbance shifted to 420 nm. The narrowing of the maximum absorption peak of P3HT_28_-*g*-PLA_72_ porous thin films and blue shift to 420 nm could be attributed both to a decrease in interchain interaction and to an increase of the amorphous polymer phase, as proven by GIWAXS measurements (Figure S4), toward a rougher film surface as shown by AFM.^68^ These results demonstrate that both non-porous and porous thin films absorb light in the visible range, making them accurate for optoceutical therapies.^69,70^ Then, the fluorescence emission properties of the thin films after excitation at 480 nm were studied. The fluorescence emission spectra of non-porous thin films show two main emission maximum peaks at 516 and 630 nm (Figure 4c). Porous thin films present similar fluorescence spectra and emission wavelengths (Figure 4d). Overall, these results demonstrate that the optical properties of porous materials do not show sizable changes in terms of spectral shifts and emission efficiency, maintaining their main absorption features, thus supporting this approach for further use as photoactive materials in the realization of bio–hybrid interfaces with living cells.

**Figure 5:**
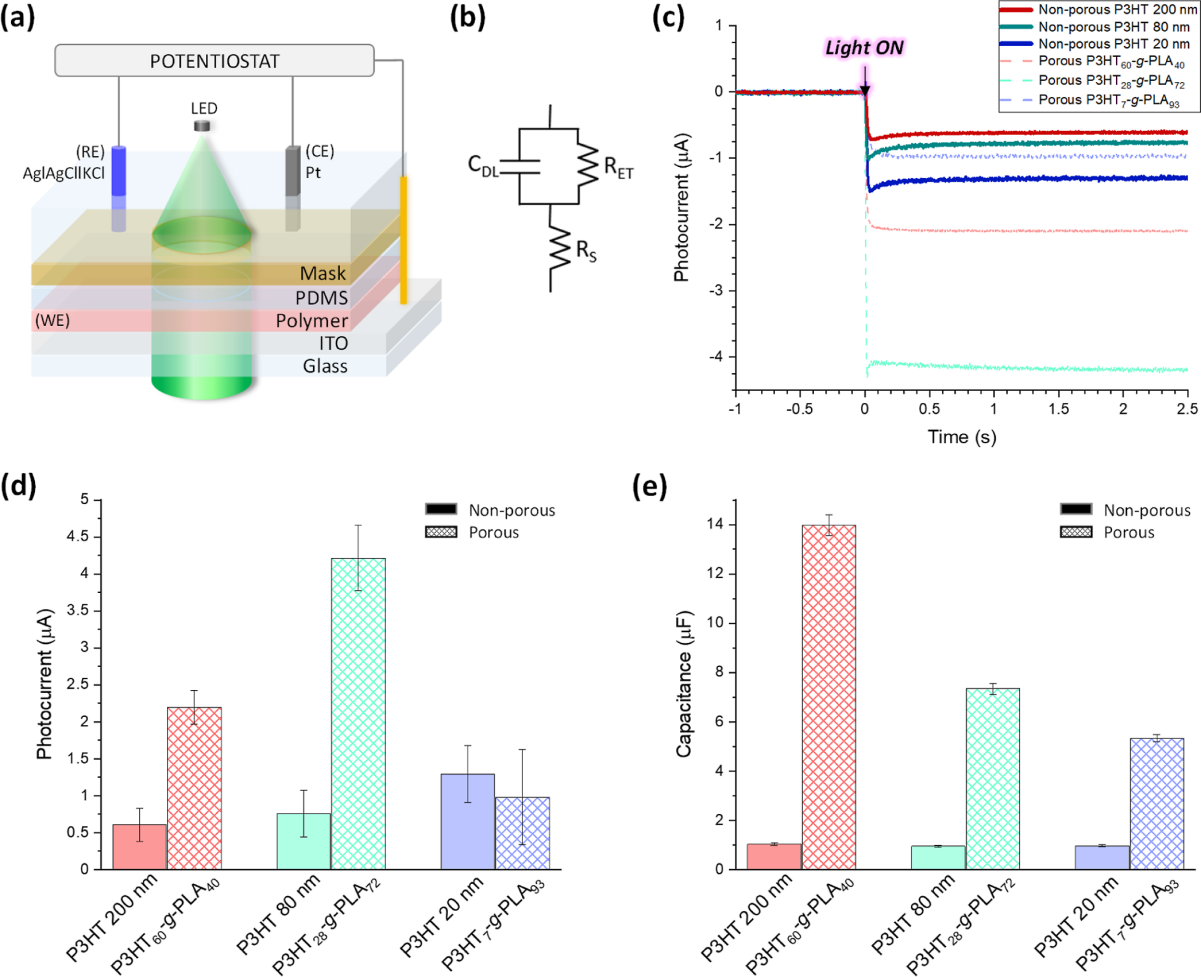
(a) Schematic representation of the PEC used for measuring the photocurrent properties and electrical capacitance. (b) Equivalent circuit model of the ITO–P3HT–PBS heterojunction used to fit EIS data. (c) Photocurrent curves of non-porous P3HT films with different thicknesses and porous films made of P3HT_7_-*g*-PLA_93_ (blue curve), P3HT_28_-*g*-PLA_72_ (green curve), and P3HT_60_-*g*-PLA_40_ (red curve) when irradiated with a LED at 110 mW cm^-2^ and 530 nm. (d) Photocurrent values after 1 s of LED irradiation of the non-porous and porous films. (e) Capacitances of non-porous and porous films as obtained from EIS, *n* = 3.

Photo-electrochemical properties were determined by using the p-type organic semiconducting films as working electrodes (WE) in contact with an aqueous electrolyte (0.1 M PBS pH 7.4) in a photoelectrochemical cell (PEC) (Figure 5a,b). The photoelectrode was connected as the WE to a potentiostat, and a Pt-wire and an Ag|AgCl|KCl (3 M) electrode were used as counter electrode (CE) and reference electrode (RE), respectively. The photoelectrode was illuminated through a quartz window and the electrolyte. Only the circular area (diameter 1 cm) of the photoelectrode in contact with the electrolyte was illuminated. To compare the data with the non-porous reference material (pure P3HT), the variation in the layer thickness has to be taken into account. We observed that the copolymers with higher PLA content resulted in thinner layers after hydrolysis (Figure S3). The experimental parameters were fine-tuned to produce non-porous and porous thin films of identical thickness for every P3HT-*g*-PLA film that underwent testing. Accordingly, we prepared reference photoelectrodes with pure P3HT of a comparable thickness (20, 80, and 200 nm). Irradiation with a green LED (λ = 530 nm, intensity = 110 mW cm^–2^, incidence from the electrolyte side) induces the quick formation of a cathodic photocurrent due to the oxygen reduction reactions (rise time < 50 ms), in line with literature reports.^39,40,71^ The photocurrent gets stable after 0.5 s of illumination (Figure 5c). The average steady-state photocurrent is shown in Figure 5d and demonstrates a 3.5-fold increase for P3HT_60_-*g*-PLA_40_ porous thin films, with pore sizes of ∼1.2 μm diameter and ∼100 nm height (Figure 2c,d), and a 5.5-fold increase for P3HT_28_-*g*-PLA_72_ porous thin films, with smaller pore sizes, ∼220 nm diameter and ∼15 nm height (Figure 2c,d). In the case of P3HT_7_-*g*-PLA_93_ films, the quantity of semiconducting polymer P3HT present in the porous film is minimum, and it is not enough to enhance the photocurrent properties.

To rationalize these findings, we performed impedance spectroscopy on illuminated photoelectrodes. The spectra were fitted using the equivalent circuit presented in Figure 5b, where *R*_S_ represents the resistance in series with the interface, *C*_DL_ is the cumulative double-layer capacity, and *R*_ET_ is the resistance accounting for the electrochemical processes occurring at the interface between the electrolyte and the semiconductor. Since the main contribution of the interface between the semiconductor and water occurs at the low end of the frequency spectrum (<10 Hz), we used this simple circuit to fit the data. To fit the overall spectrum over a broader frequency range, we used a more complex circuit that also accounts for the ITO–semiconductor interface (Figure S8a). The determined thin film capacitances are shown in Figure 5e. For the non-porous thin films, we observe that the capacitance is nearly independent of thickness. Instead, in porous thin films, there is a steady increase in capacitance with thickness. A similar finding is also observed for the charge-transfer resistance, which shows a decrease with increasing thickness of the porous films (Figures S8b,c and S9).

These observations demonstrate that the considerable increase in photocurrent and capacitance of the porous thin films is attributed to the larger active surface area between the polymer and the electrolyte caused by the porosity. Consequently, an increased number of dissolved oxygen molecules comes in close contact with excited electrons located in the polymer and induces increased charge transfer. Overall, the P3HT_28_-*g*-PLA_72_ films show the best photocurrent properties and were selected for further *in vitro* cell assays.

### 2.3 *In Vitro* Cytotoxicity Assay and ROS Production

**Figure 6:**
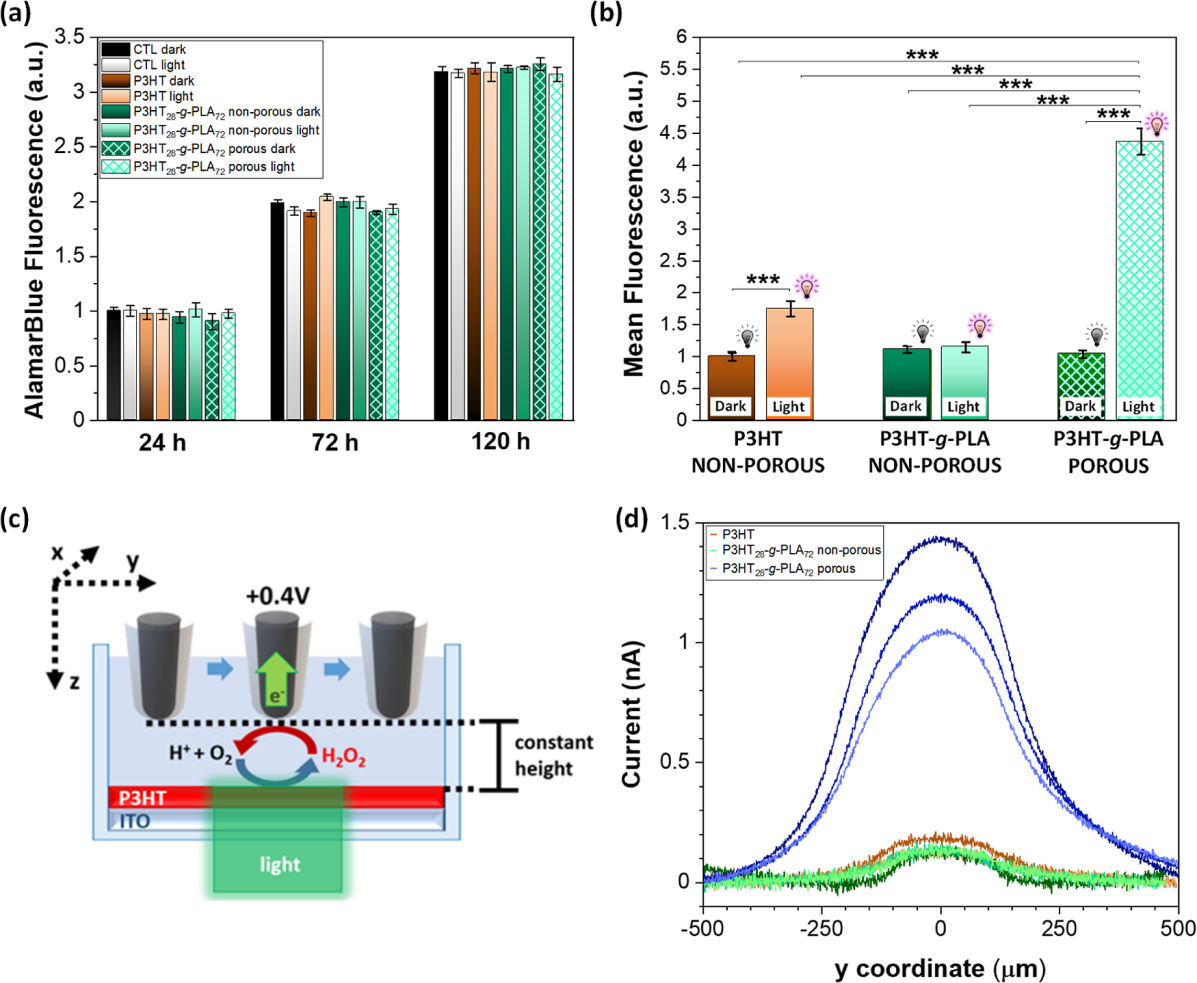
(a) *In vitro* proliferation assay of HUVECs plated on P3HT, non-porous (solid bars), and porous (frame filler bars) P3HT_28_-*g*-PLA_72_ films up to 120 h. (b) *In vitro* ROS production test of non-porous (solid bars) and porous (frame filler bars) P3HT_28_-*g*-PLA_72_ films in contact with HUVECs cells in the darkness and after irradiation with a LED light (λ_exc_ = 520 nm; 110 mW cm^–2^) for 2.5 s. Results are shown as mean ± s.e.m. (3 biological replicates, *n* = 9 samples per each condition, *m* = 900 cells per each condition) with statistical tests performed by ANOVA with Bonferroni correction at a significance level of ***p < 0.001. (c) Schematic representation of the SECM setup for the quantitative measurement of photo-induced ROS production at the polymer/electrolyte P3HT/PBS interface. (d) Oxidation currents of H_2_O_2_ measured at a black platinum working microelectrode at 0.4 V *vs* Ag/AgCl (KCl 3 M) for P3HT thin films (brown curves), non-porous (green curves), and porous (blue curves) P3HT_28_-*g*-PLA_72_ thin films, *n* = 3.

Cell fate, in terms of adhesion, proliferation, and migration processes, is precisely regulated by intracellular redox state and the capability to produce/quench ROS. Thus, we directly addressed the performance of the polymer porous photoelectrodes to regulate intracellular ROS production by using them as cell-culturing substrates in *in vitro* studies. As a relevant biological model, we chose endothelial HUVECs since they play central roles in both cardiac remodeling and regeneration.^72,73^ First of all, the proliferation of cells cultured on top of films was evaluated for up to 5 days (Figure 6a). Results show no cytotoxic effect in either dark or light conditions, with effective cell proliferation up to 120 h after plating. Intracellular ROS production was investigated by fluorescence microscopy by incubating HUVECs with the 2′,7′-dichlorodihydrofluorescein diacetate (H_2_-DCF-DA) probe (Figure 6b and Figure S10). Data have been normalized to the reference control condition, *i.e.*, non-porous P3HT film. As expected, in dark conditions, all samples showed similar ROS production activity. Upon illumination, non-porous P3HT film showed a statistically significant ROS increase, in line with previous reports.^50^ Conversely, no increase with respect to dark conditions was observed in the case of non-porous P3HT_28_-*g*-PLA_72_ films. This fact could be attributed to the lower photocurrent intensity exhibited by non-porous P3HT_28_-*g*-PLA_72_ films in comparison to pure P3HT films with the same thickness (Figure S11). Most importantly to this work, the porous P3HT_28_-*g*-PLA_72_ film exhibited a >4-fold increase in ROS production upon irradiation, thus confirming the close interplay among the material porosity, the increase of the surface area available for photoelectrochemical reactions, the higher photocurrent density, and the modulation of intracellular ROS species. In order to quantitatively evaluate photo-induced H_2_O_2_ production at the film/electrolyte interface as well as to provide information about its spatial distribution, we carried out scanning electrochemical microscopy (SECM) measurements (Figure 6c). The 10 μm SECM probe was positioned at approximately 20 μm from the surface of the P3HT thin film, this distance being comparable to cellular dimensions. As a consequence, photo-induced ROS concentration increase was monitored at a distance from the electrolyte/polymer interfaces, which better represents the extracellular environment that is experienced by cells adhering on top of the polymer thin films. High sensitivity and specificity of the probe toward ROS detection were obtained by employing black platinum-modified microelectrodes^40,74,75^ (see the experimental section for details on the modification of the micrometric probe with nanoporous black platinum). To characterize the proficiency of the different types of P3HT thin films to locally change the ROS levels, photostimulation of the thin film surface was restricted to a circular area with a diameter of approximately 100 μm (see the experimental section for a detailed description of the experimental apparatus). Local ROS concentrations in the vicinity of the stimulating light spot were determined by scanning the probe over a region that crosses the illuminated portion of the P3HT thin film and by keeping the probe at a constant height (20 μm) from the P3HT thin film surfaces (as schematized in Figure 6c). The oxidation currents (Figure 6d) are due to the electrochemical oxidation of ROS species at the black platinum probe, and the current signals are quantitatively related to the local ROS concentrations: the current signals are proportional to the amount of H_2_O_2_ generated at the film/electrolyte interface [Marcu, 2012; Malferrari, 2019].^76,77^Figure 6d shows the current scan curves, which report on the H_2_O_2_ levels, which are recorded by the microelectrode as a function of the linear displacement along the *x*-direction of the scanning WE and that crossed the light spot at its centers in the *y* directions. The recorded intensity profiles closely follow the Gaussian profile of the excitation beam. The intensity of the H_2_O_2_ oxidation currents considerably increased in the case of P3HT_28_-*g*-PLA_72_ porous thin films in comparison with non-porous P3HT_28_-*g*-PLA_72_ and P3HT films, leading to an 8-fold increase in the concentration of H_2_O_2_ that reached values of 8 μM (Table 2) for the specific illumination powers and wavelengths employed in this setup. These values are superior to those reported for P3HT solid films that exhibited 0.7 μM H_2_O_2_ production (those measurements were accomplished in the same setup as for the ones shown herein).^40^

**Table 2:** Average Currents from ROS Oxidation at Black Platinum-Modified Microelectrodes and Maximal H_2_O_2_ Concentration Estimated

thin film	morphological structure	H_2_O_2_ oxidation currents (nA)	[H_2_O_2_] (μM)
P3HT	non-porous	0.148 ± 0.024	1.1 ± 0.4
P3HT_28_-*g*-PLA_72_	non-porous	0.133 ± 0.012	1.0 ± 0.2
P3HT_28_-*g*-PLA_72_	porous	1.213 ± 0.205	8.0 ± 1.0

Overall, these data confirm that porous thin films, while do not have a negative impact on HUVEC adhesion and proliferation, induce a sizable increase in intracellular ROS production, thanks to the maximization of the interfacial area available for photoelectrochemical reactions.

## 3 Conclusions

Herein, we reported the use of nanoporous semiconducting polymer films as photoelectrodes for ROS generation. For this purpose, we developed a methodology to obtain nanoporous P3HT thin films based on the selective hydrolysis of the polyester segment of a graft copolymer. The synthesis of hydrolyzable p-type semiconducting graft polymers, P3HT-*g*-PLA, was carried out by chemical oxidative polymerization of 3HT and a specific EDOT-PLA macromonomer obtained by ROP under controlled conditions. Then, thin films of the graft copolymers were prepared by spin coating, and next the porosity was induced by selective PLA graft hydrolysis in the presence of NaOH. The porosity of the P3HT was modulated from ∼220 nm to 1.2 μm by the graft copolymer composition as determined by AFM.

Interestingly, the porous P3HT films exhibited similar optical absorption properties to bulk P3HT films but a 5.5-fold enhancement in photocurrent generation. This effect has a positive impact on the optical ability of the p-type semiconducting porous thin films to modulate the intracellular ROS concentration of HUVEC cells more effectively, leading to a 4.5-fold increase in the intracellular ROS production of porous films in comparison to non-porous films. Hence, it provides a promising achievement for the fabrication of wireless optoelectronic devices that open the way to future optoceutical therapies for cardiac tissue regeneration.

## 4 Experimental Section

### 4.1 Materials

3-hexylthiophene ≥ 98.0% (3HT) and hydroxymethyl EDOT ≥ 97.0% were supplied by TCI; iron(III) chloride, sodium hydroxide, and methanol ≥ 99.9% were purchased from Fluka; methanesulfonic acid ≥ 99.0% ≥ 99.0% (MSA) and 4-(dimethylamino)pyridine ≥ 98.0% (DMAP) were provided by Sigma-Aldrich and used as received. l-Lactide ≥ 85.0% (LA) was purchased from Sigma-Aldrich and purified by solving the lactic acid in toluene at 60 °C, followed by the precipitation and crystallization at room temperature twice. Then, the toluene was removed by rotary evaporation, and the final product was dried under vacuum.

### 4.2 Synthesis of PLA Macromonomer

The α-EDOT-PLA macromonomer with a molecular weight of 13,000 g mol^–1^ was synthesized by ROP of l-lactide in bulk using hydroxymethyl EDOT as chain initiator and a mixture of MSA and DMAP (2MSA/3DMAP) as organocatalyst. First, the organocatalyst mixture was heated up at 100 °C until forming a white salt. Then, the l-lactide and hydroxymethyl EDOT were added and left to react at 130 °C under magnetic stirring in an inert atmosphere for 90 min until a 90% conversion was achieved. The resulting product, EDOT-PLA, was purified through precipitation in methanol and vacuum-dried at room temperature overnight.

### 4.3 Synthesis of P3HT and P3HT-*g*-PLA Copolymers

P3HT was synthesized by chemical oxidative copolymerization of 3HT using FeCl_3_ as an oxidizing agent (4 equiv respect 3HT monomer) and acetonitrile as solvent at room temperature overnight. Graft copolymers with different compositions were synthesized by chemical oxidative copolymerization of 3HT and the α-EDOT-PLA macromonomer using FeCl_3_ (4 equiv respect 3HT monomer) as an oxidizing agent and acetonitrile as a solvent. The reaction was carried out under magnetic stirring at room temperature overnight. In all cases, the dark brown dispersions obtained were precipitated in methanol, rinsed with methanol and water until the iron residue was fully removed, and dried under vacuum.

### 4.4 Proton Nuclear Magnetic Resonance Spectroscopy (^1^H NMR)

^1^H NMR spectra were recorded with a Bruker ADVANCE DPX 300 at 300.16 MHz resonance frequency, room temperature, and using CDCl_3_ as solvent. The experimental conditions were: 10 mg of sample, 3 s acquisition time, 1 s delay time, 8.5 μs pulse, spectral width of 5000 Hz, and 32 scans.

### 4.5 Gel Permeation Chromatography or Size Exclusion Chromatography

The molecular weight was determined with a Waters GPC instrument, equipped with one guard column and three gel permeation columns in series (Styragel HR2, HR4, and HR6), and a refraction index detector (Waters 2414), and a Waters 717 plus autosampler. THF was used as an eluent at a flow rate of 1.0 mL min^–1^, and the relative molecular weight was determined through a conventional calibration obtained with PS narrow standards, ranging from 578 to 3,147,000 g mol^–1^.

### 4.6 Film Preparation

Films were prepared on ITO-glass substrates of (7.5 × 10 mm, Ossila), previously cleaned with acetone and isopropanol, using a spin coater (Ossila). First, non-porous thin films were prepared by spin-coating 20 μL of P3HT or P3HT-*g*-PLA solutions at a concentration of 40 mg mL^–1^ in chlorobenzene over the ITO-glass substrate at 3000 rpm for 60 s. Porous films were obtained in a second step by PLA hydrolysis in 0.5 M NaOH for 30 min, followed by a rinsing step with water, and finally dried at room temperature.

### 4.7 Transmission Electron Microscopy and Energy Dispersion X-ray Analysis

The structure and morphology of non-porous and porous films were visualized by TEM using a Talos F200i field emission gun instrument equipped with a Bruker X-Flash100 XEDS spectrometer. Elemental maps were performed by XEDS in the scanning TEM (STEM) mode under a high annular dark field detector for *Z* contrast imaging in STEM conditions (camera length of 160 mm) using a pixel size of 28 nm, a dwell time of 900 s, and an image size of 512 × 512 pixels. Moreover, EDX microanalyses were carried out using a probe current of 500 pA and a semiconvergence angle of 8.5 mrad. For TEM measurements, non-porous films were prepared over silicon substrates (7.5 × 10 mm) coated with polystyrene sulfonate (PSS). Then, films were detached from silicon substrates by immersion in Milli-Q water, deposited over carbon-coated copper grids, and dried at room temperature before observation. Subsequently, porous films were obtained by depositing a drop of 0.5 M NaOH over the films placed on the carbon-coated copper grids. After 30 min, the samples were rinsed with Milli-Q water and dried at room temperature before observation. TEM images were analyzed with the software ImageJ.

### 4.8 Grazing-Incidence Wide-Angle X-ray Scattering (GIWAXS)

X-ray scattering measurements were performed at the NCD-SWEET beamline at the ALBA Synchrotron (Barcelona, Spain) and at the BM28-XMaS beamline at the European Synchrotron (Grenoble, France). Regarding the NCD beamline, we used a Rayonix LX255-HS to collect the 2D patterns. At XMaS, we used a MarCCD 165. For both beamlines, the energy was set at 12.4 keV; we used acquisition times below 10 s and incident angles around 0.12°.

### 4.9 Atomic Force Microscopy

The morphology of non-porous and porous films was analyzed by AFM using a multimode microscope, controlled using NanoScope V electronics (Bruker), and running Nanoscope 8.15 software (Build R3Sr8.103795). Before AFM measurements, the films prepared on ITO-glass substrates were dried in a vacuum chamber overnight. Analysis of the AFM images was carried out using the Nanoscope Analysis 1.90 software (Bruker). The surface area of the films was determined from the AFM height images (5 μm × 5 μm) by measuring the pore diameters and heights with the Software NanoScope Analysis 1.90 and using the following equation
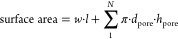
1where *w* and *l* are the width and length of the film, respectively; *N* is the number of pores; and *d*_pore_ and *h*_pore_ are the pore diameter and pore height, respectively.

### 4.10 Contact Angle

The surface wettability of the films was determined by static WCA measurements through the sessile drop method with a standard OCA 20 goniometer (DataPhysics) and the SCA 20 software under ambient conditions. A water droplet of 3 μL was deposited on the films on ITO-coated glass substrates, and the contact angle was measured from recorded pictures with the SCA 20 software. Four different samples were measured, and the results are shown as the mean ± standard deviation.

### 4.11 UV–Vis Spectroscopy

Absorbance properties of non-porous and porous films prepared on ITO-glass substrates were analyzed by UV–Vis spectroscopy. UV–vis spectra from 350 to 700 nm were recorded with a Shimadzu UV-2550 spectrometer equipped with a film adapter.

### 4.12 Fluorescence Spectroscopy

Fluorescence emission spectra of non-porous and porous films prepared on ITO-glass substrates were recorded from 510 to 800 nm after exciting the samples at a wavelength of 480 nm using a PerkinElmer LS 55 Fluorescence Spectrometer.

### 4.13 Photocurrent Measurements and Electrochemical Impedance Spectroscopy

The active surface of the photoelectrode was exposed to PBS electrolyte (0.01 M phosphate buffer, 0.137 M NaCl). By mounting the photoelectrode in a dedicated measurement cell, only the semiconducting layer is exposed to the electrolyte (through a 1 cm diameter mask), while the buried ITO surface and the electrical contacts remain separated *via* a polydimethylsiloxane O-ring. The measurement cell allows the exposition to the illumination from either the ITO side or the solution side, the latter through a quartz window. In a three-electrode setup, the photoelectrode was operated as the WE. An Ag|AgCl (3 M KCl) RE was used in combination with a Pt CE. The light source was a monochromatic LED (Thorlabs M530L4) driven by a source-measure unit (Thorlabs DC2200). A potentiostat (Metrohm PGSTAT204) was used to set the voltage and collect the current. The potentiostat was also used for impedance spectroscopy measurements. Results are shown as mean ± standard deviation (*n* = 3 samples).

### 4.14 Cell Culture

HUVECs were purchased from PromoCell and grown in endothelial cell basal medium (PromoCell), supplemented with endothelial cell GM 2 supplement pack (PromoCell). Cells were kept in T-75 culture flasks coated with 0.2% gelatin and maintained in an incubator at 37 °C in a humidified atmosphere with 5% CO_2_. For the experiments, only HUVECs at passage < 7 were employed. After reaching 80–90% of confluence, cells were detached by incubation with 0.5% trypsin–0.2% EDTA (Sigma-Aldrich) for 5 min and then plated on the different samples for experiments.

### 4.15 AlamarBlue Proliferation Assay

Prior to cell plating, a layer of 1 mg mL^–1^ fibronectin (from bovine plasma, Sigma-Aldrich) in PBS (Sigma-Aldrich) was deposited on the surface of the samples and incubated for 30 min to promote cell adhesion. After washing with PBS, cells were plated onto P3HT-based samples in 12-well plates at about 10^4^ cells cm^–2^ density. Cell proliferation was evaluated after 24, 48, and 120 h from plating by considering 2 biological replicates. To this aim, AlamarBlue cell reagent was diluted 1:10 in the cell culture medium. Three aliquots of culture media for each condition were placed in a black 96-well microplate, and the fluorescence of the AlamarBlue compound was acquired by a plate reader (TECAN Spark 10M Plate Reader) with an excitation/emission wavelength of 540/600 nm. The procedure was repeated at each time point, rinsing, and replacing the AlamarBlue compound with fresh medium after each measurement.

### 4.16 ROS Production

H_2_DCF-DA (purchased from Sigma-Aldrich) was employed for intracellular detection of ROS. HUVECs were cultured on P3HT-based substrates (10^4^ cells cm^–2^). The samples in light condition were photo-excited by illuminating each sample for 2.5 s with an LED system (Thorlabs, λ = 520 nm, 110 mW cm^–2^). The samples were illuminated from the ITO side. Subsequently, cells were incubated with H_2_DCF-DA for 30 min (10 μM) in Krebs Ringer’s (KRH) extracellular solution (mM): 135 NaCl, 5.4 KCl, 1.8 CaCl_2_, 1 MgCl_2_, 5 HEPES, 10 glucose, pH adjusted to 7.4 with NaOH. After careful wash-out of the excess probe from the extracellular medium, the fluorescence of the probes was recorded (excitation/emission wavelengths, 490/520 nm; integration time, 500 ms; binning: 1 × 1) with an upright microscope (Olympus BX63) equipped with a 20× objective and an sCMOS Camera (Prime BSI, Teledyne Photometrics; Tucson, Arizona, USA). Variation of fluorescence intensity was evaluated over regions of interest covering single cell areas. Reported values represent the average over multiple cells (*n* > 900) belonging to 9 statistically independent samples tested in 3 different experimental sessions. Image processing was carried out with ImageJ. Origin Pro 2018 was employed for data analysis.

### 4.17 Scanning Electrochemical Microscopy

Scanning electrochemical measurements were carried out with a CHI910B SECM bipotentiostat from CH Instruments Inc (Austin Texas) and by employing a three electrode setup; the piezoelectric and stepper components of the SECM instrument were mounted on the plate of a Nikon Ti microscope, thus enabling optical imaging of the same area imaged by SECM. Platinum microelectrodes were modified with nanoporous black platinum as described earlier^77^ and used as WEs; a Ag/AgCl (KCl 3 M) electrode and a platinum wire were used as REs and CEs, respectively. Photoinduced ROS production was studied with P3HT thin films immersed in PBS medium (Gibco, part of Thermo Fisher, Paisley, United Kingdom, product no. 4040091) as an electrolyte. Spatially controlled illumination was achieved as detailed earlier^40^ with a mercury lamp of an inverted fluorescence Nikon Ti microscope filtered with a Nikon Texas Red HYQ cubic filter (excitation, 532–587 nm; emission, 608–683 nm). The power density, measured at 550 nm with an optometer at its focal plane, was approximately 20 mW mm^−2^.

## Supporting Information Available

GPC spectra and Mn of the copolymers; ^1^H NMR spectra of the thin films after PLA hydrolysis; thickness of non-porous and porous thin films measured by AFM; GIWAXS measurements of non-porous and porous thin films; 3D AFM images of porous films; AFM images of PLA films before and after hydrolysis; WCA of non-porous and porous thin films; fluorescence images of HUVEC cells cultured on top of thin films; optical absorption and photoelectrochemical current spectra of photoelectrode thin films; Nyquist plots and impedance curves and the corresponding fits for non-porous and porous thin films (PDF)
