# Supplementary material for: Semiconducting Polymer Nanoporous Thin Films as a Tool to Regulate Intracellular ROS Balance in Endothelial Cells
Source: ACS Appl Mater Interfaces. 2023 Jul 19;15(30):35973–85. doi: 10.1021/acsami.3c06633 (PMC10401575; doi:10.1021/acsami.3c06633)
Supplement: Supplementary file 1 — am3c06633_si_001.pdf [file am3c06633_si_001.pdf]

Supporting Information

Semiconducting polymer nanoporous thin films as a tool to regulate intracellular ROS balance in endothelial cells

Miryam Criado-Gonzalez,[1] Luca Bondi,[2] Camilla Marzuoli,[3,4] Edgar Gutierrez-Fernandez,[1,5] Gabriele Tullii,[3] Carlotta Ronchi,[3] Elena Gabirondo,[1] Haritz Sardon,[1] Stefania Rapino,[6] Marco Malferrari,[6] Tobias Cramer,[2] Maria Rosa Antognazza,[3] and David Mecerreyes[1,7]**

[1] POLYMAT, University of the Basque Country UPV/EHU, Paseo Manuel de Lardizabal 3, 20018 Donostia-San Sebastián, Spain

[2] Department of Physics and Astronomy, University of Bologna, Viale Carlo Berti Pichat 6/2, 40127 Bologna, Italy

[3] Center for Nano Science and Technology@PoliMi, Istituto Italiano di Tecnologia, Via Raffaele Rubattino 81, 20134 Milano, Italy

[4] Politecnico di Milano, Dipartimento di Fisica, Piazza Leonardo da Vinci 32, 20133 Milano, Italy

[5] XMaS/BM28-ESRF, 71 Avenue Des Martyrs, F-38043 Grenoble Cedex, France

Department of Physics, University of Warwick, Gibbet Hill Road, Coventry, CV4 7AL, UK

[6] Department of Chemistry "Giacomo Ciamician", University of Bologna, 40126 Bologna, Italy

[7] Ikerbasque, Basque Foundation for Science, 48013 Bilbao, Spain

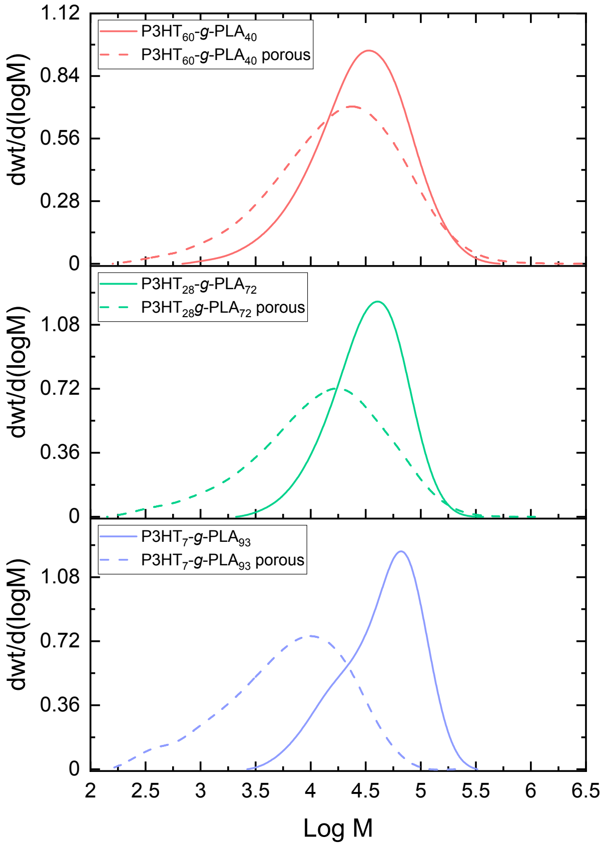

Figure S1. Molar mass distribution curves of the synthesized copolymers before after PLA hydrolysis determined by SEC.

Table S1. Number-average molecular weight (M_n) of the synthesized copolymers after PLA hydrolysis.

Code	Synthesized polymer	M_n (g mol^{-1})[a]	D[b]
2	$P3HT_{60}$-*g*-PLA_{40} porous	6 300	6.2
3	$P3HT_{28}$-*g*-PLA_{72} porous	4 400	5.9
4	$P3HT_{7}$-*g*-PLA_{93} porous	2 600	4.4

[a)]M_n determined by using PS standards; [b)]Dispersity (D) = M_w/M_n calculated by SEC.

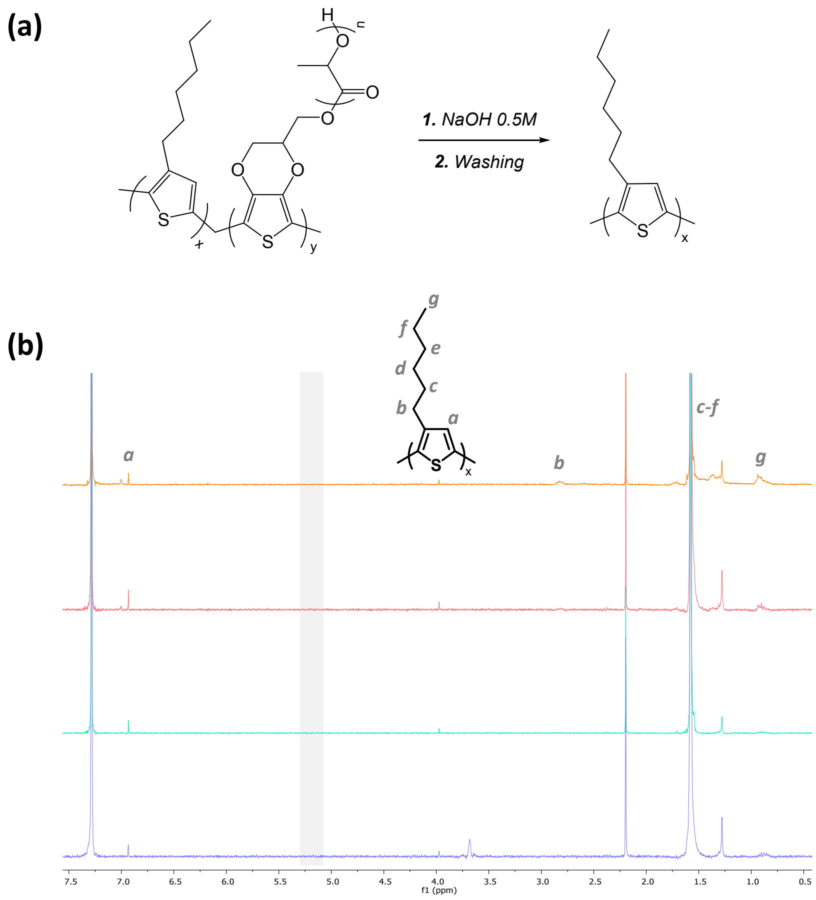

Figure S2. (a) Chemical route employed to hydrolyze PLA. (b) ^{1}H-NMR spectra of the thin films, P3HT (orange curve), P3HT$_{60}$-*g*-PLA$_{40}$ (red curve), P3HT$_{28}$-*g*-PLA$_{72}$ (green curve), and P3HT$_{7}$-*g*-PLA$_{93}$ (blue curve), after NaOH treatment, to induce the PLA hydrolysis.

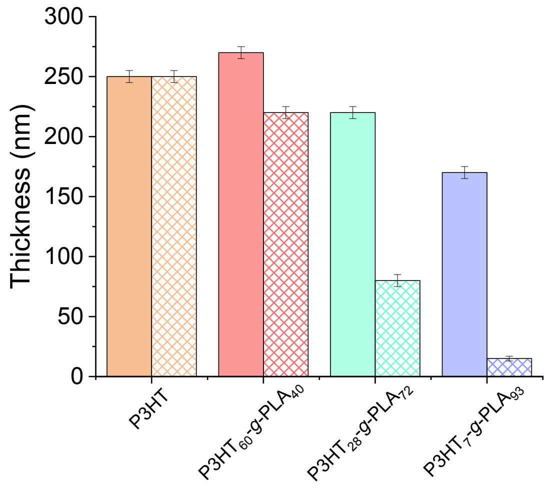

Figure S3. Thickness of the non-porous (solid bars) and porous (frame filler bars) films measured by AFM. (b) Water contact angle (WCA) of non-porous (solid bars) and porous (frame filler bars) films.

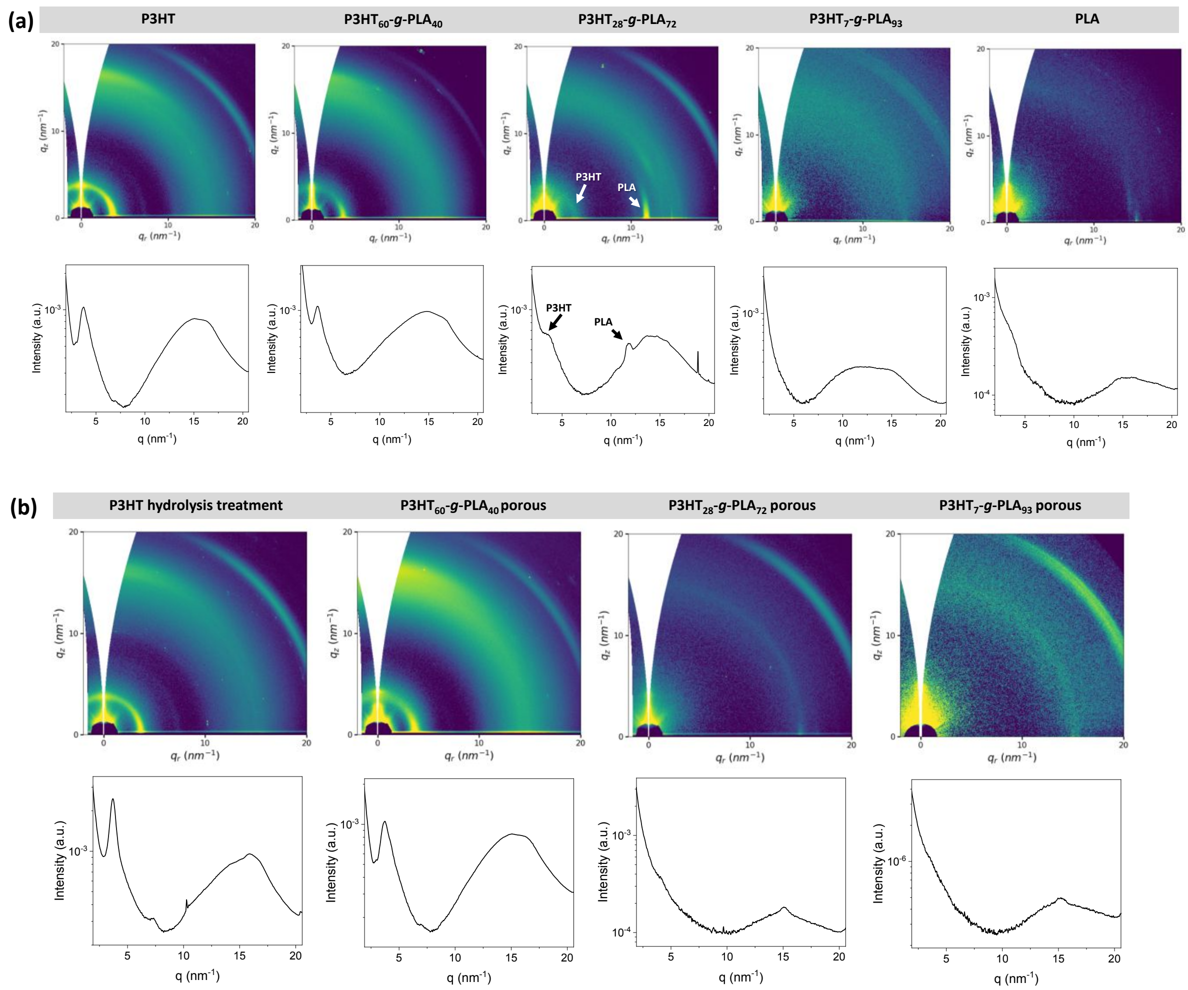

Figure S4. 2D GIWAXS images and the corresponding Intensity *vs.* *q* curves plots for the (a) non-porous films made with the homopolymers, P3HT and PLA, and the different graft copolymers P3HT-*g*-PLA, and (b) porous thin films.

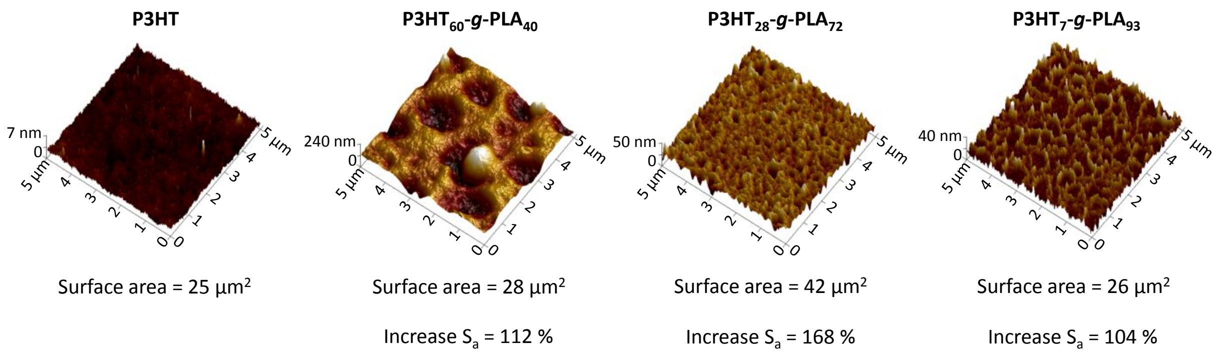

Figure S5. 3D AFM height images of porous films and surface area (S_a) determined with the software Nanoscope Analysis 1.90 and the Equation 1. The increase S_a was determined with respect to the surface area of P3HT film.

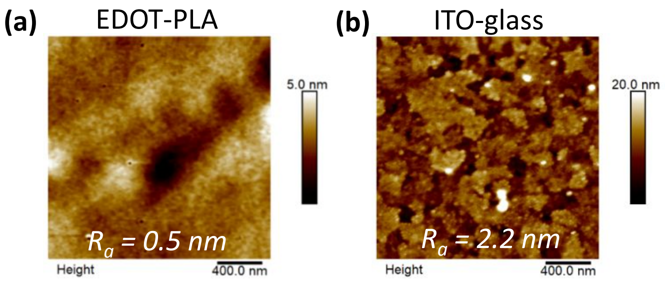

Figure S6. Topographical AFM images of the (a) non-porous PLA films and (b) ITO-glass after the complete PLA hydrolysis.

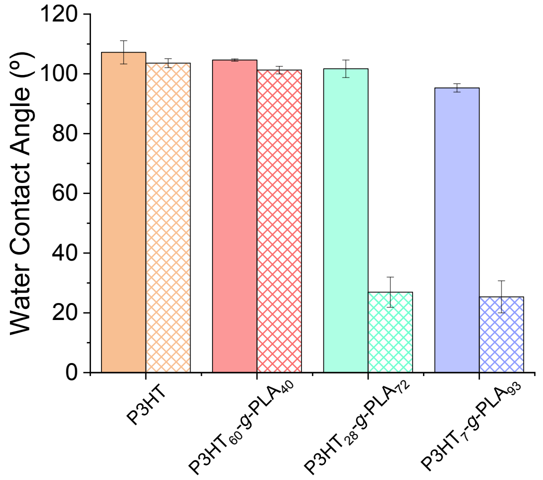

Figure S7. Water contact angle (WCA) of non-porous (solid bars) and porous (frame filler bars) films.

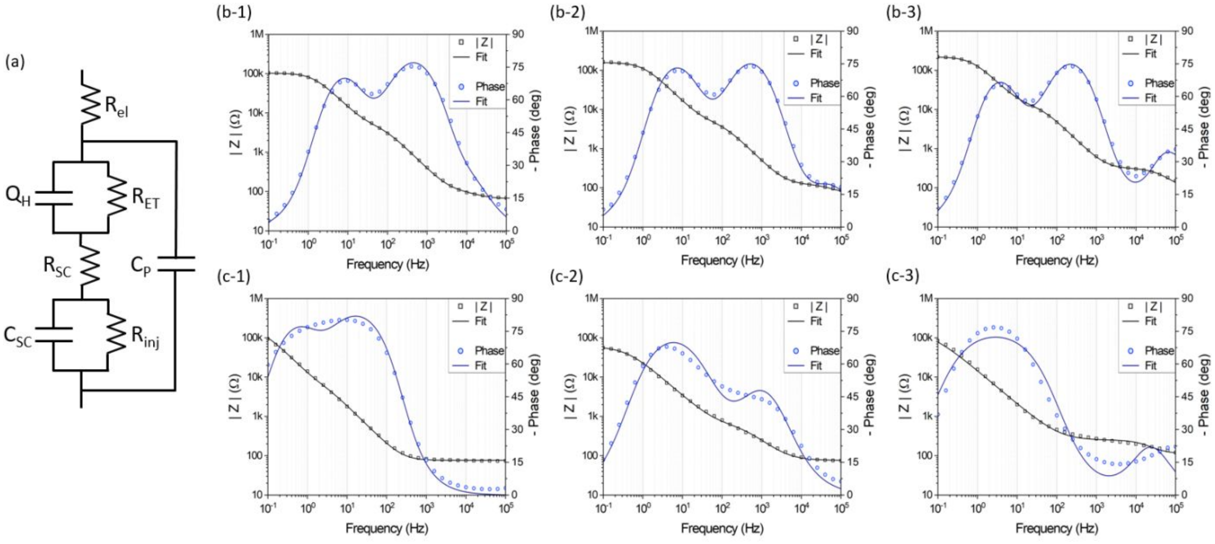

Figure S8. (a) Schematic representation of the extended equivalent circuit used to fit the impedance data between 100 mHz and 100 kHz. The circuit includes several elements, namely R_{inj} and C_{SC}, representing the injection resistance and the capacitance at the ITO-semiconductor interface, R_{SC}, the semiconductor resistance that becomes important in thick films, Q_H, the non-ideal double-layer capacitance which is placed in parallel with the electron transfer resistance R_{ET} at the interface with water, R_{el}, the electrolyte resistance, and C_P, the parallel capacity accounting for the unbalanced charges between ITO and the electrolyte. The impedance curves of the samples and the corresponding fits for non-porous (b) and porous (c) samples are shown as empty symbols and straight lines, respectively. The samples are ordered by thickness: 20 nm (1), 80 nm (2), and 200 nm (3).

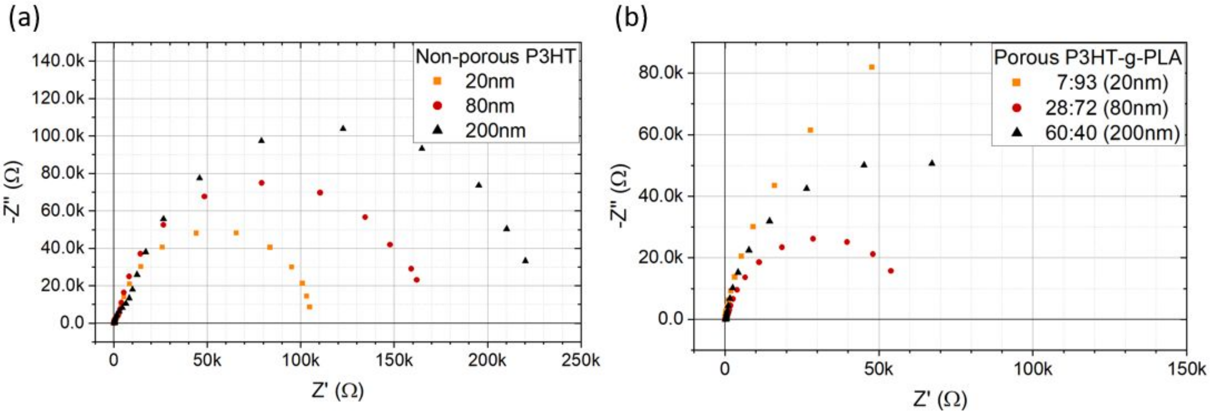

Figure S9. Nyquist plots showing impedance data acquired from 100 mHz to 100 kHz for non-porous (a) and porous (b) films.

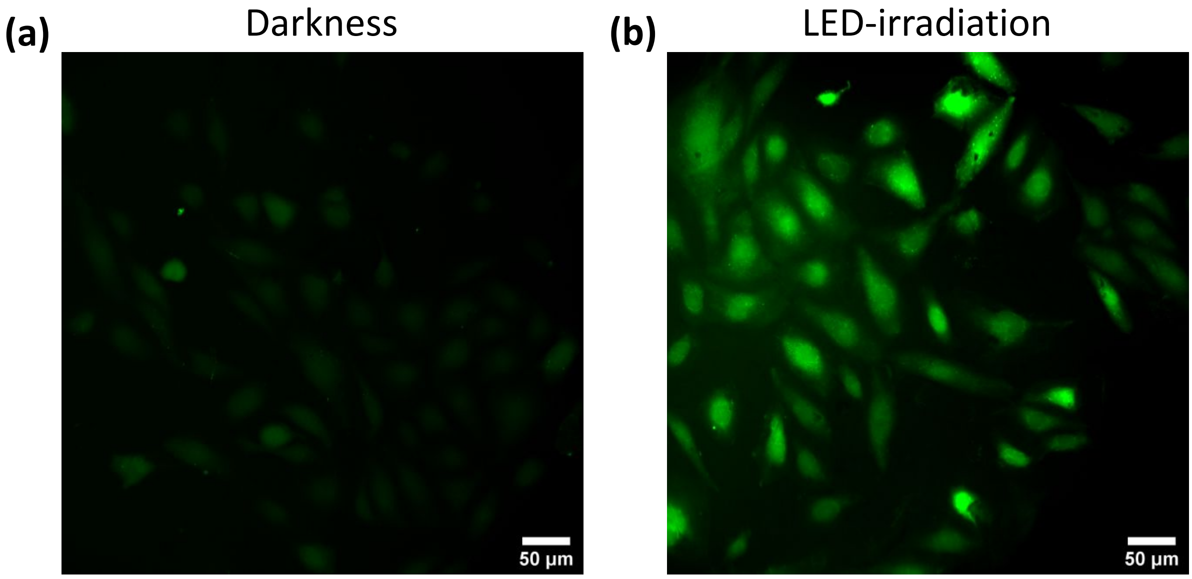

Figure S10. Fluorescence images of HUVEC cells cultured on top of the P3HT$_{28}$-*g*-PLA$_{72}$ porous thin films (a) in the darkness and (b) after irradiation with a LED (λ_{exc} = 520 nm; 110 mW cm^{-2}) for 2.5 s.

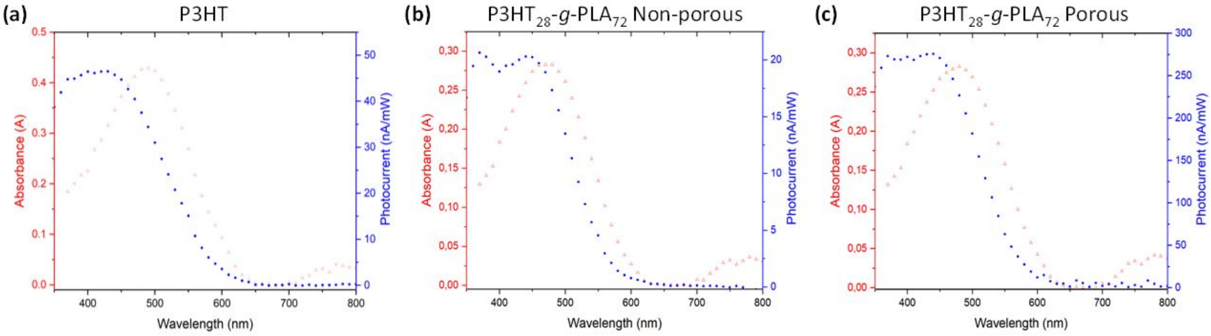

Figure S11. Optical absorption and photoelectrochemical current spectra of the three different photoelectrode thin films (a) P3HT, (b) Non-porous P3HT$_{28}$-*g*-PLA$_{72}$, and (c) Porous P3HT$_{28}$-*g*-PLA$_{72}$ in contact with the electrolyte (PBS at pH 7.4).
